# Efficacy of Specific Trunk Exercises in the Balance Dysfunction of Patients with Parkinson’s Disease: A Systematic Review and Meta-Analysis

**DOI:** 10.3390/s23041817

**Published:** 2023-02-06

**Authors:** Remedios López-Liria, Sofía Vega-Tirado, María Ángeles Valverde-Martínez, Andrés Calvache-Mateo, Ana María Martínez-Martínez, Patricia Rocamora-Pérez

**Affiliations:** 1 Health Research Centre, Department of Nursing, Physiotherapy and Medicine, University of Almería, Carretera del Sacramento s/n, 04120 La Cañada de San Urbano, Almería, Spain; 2 Hum-498 Research Team, University of Almería, 04120 La Cañada de San Urbano, Almería, Spain; 3 Department of Physiotherapy, Faculty of Health Sciences, University of Granada, Av. De la Ilustración 60, 18016 Granada, Granada, Spain; 4 Department of Education, University of Almería, 04120 La Cañada de San Urbano, Almería, Spain

**Keywords:** Parkinson’s disease, trunk exercises, physical therapy

## Abstract

Parkinson’s disease (PD) is a neurodegenerative pathology classified as a movement disorder. Physical exercise within a physiotherapy program is an important element to improve postural stability, balance and mobility in order to reduce falls in people with PD. The aim of this work was to determine the efficacy of specific balance and trunk mobility exercises, as well as their benefits for and effects on patients with idiopathic PD. A systematic review and meta-analysis was conducted according to PRISMA standards. The search was performed in five databases: Cochrane Library, SciELO, PEDro, Scopus and PubMed, in February 2022 with the following descriptors: Parkinson’s disease, trunk, exercise, therapy and physical therapy. The inclusion criteria were randomized controlled trials (RCTs) over the last ten years. A meta-analysis on static and dynamic balance was conducted with the software Review Manager. Nine articles met the objectives and inclusion criteria, with a total of 240 participants. The trials had moderate methodological quality according to the PEDro scale. The studies included differed with regard to intervention protocol and outcome measures. Finally, eight studies were included in a quantitative analysis in which it was shown that trunk-specific exercises interventions did not significantly improve static balance (SMD = −0.10, 95% CI = −0.29, 0.08; *p* = 0.28) or dynamic balance (SMD = 0.64 95% CI = −0.24, 1.52; *p* = 0.15). However, significant differences were found in static balance measured subjectively using the Berg Balance Scale (SMD = −0.52, 95% CI = −1.01, −0.02; *p* = 0.04). Although some differences were not significant, the studies included in this systematic review consider that specific trunk exercises or balance training combined with muscle strengthening in patients with idiopathic PD should be a complement to pharmacological treatment for improving balance dysfunction and postural instability, preventing falls and promoting wellness.

## 1. Introduction

Parkinson’s disease (PD) is a neurodegenerative pathology classified as a movement disorder [1,2,3]. The etiology is still not well defined; it is complex and involves both environmental and genetic factors (5–10%). In recent years, it has been determined that both old age and being male are the factors that most influence an increased risk of suffering from PD [1,4].

Parkinson’s disease is the second most prevalent neurodegenerative disease among the adult population following Alzheimer’s. By 2040, it is forecasted that more than 12 million people in the world will be affected by PD, having a severe impact not only on their quality of life, but also economically, as a result of the healthcare resources devoted to this issue [5,6]. The average survival time is 11 to 15.8 years, the main causes of death being pneumonia (11–28%), cardiovascular diseases (12–19%) and cancer (12–14%) [6].

There is no test capable of distinguishing PD from other disorders with similar clinical symptoms; its diagnosis is mainly based on clinical criteria [1,7]. The most widely accepted diagnostic criteria were introduced by the United Kingdom (UK) Parkinson Disease Society—Brain Bank, including four cardinal signs: bradykinesia–akinesia, resting tremor, rigidity and postural instability [8]. However, in 2015, the International Parkinson and Movement Disorder Society (MDS) proposed nine criteria, recognizing non-motor symptoms (NMS) as essential concepts related to the disease [9,10].

PD patients can display motor symptoms (MS) such as bradykinesia, rigidity, tremors, gait freezing, movement disorders, postural instability, abnormal axial posture, axial rigidity and NMS (psychiatric symptoms, depression, dementia, psychosis) [9,10]. These symptoms do not develop until approximately 50–60% of the nigral neurons are lost and about 80–85% of the dopamine content in the corpus striatum has demised [1,11].

As the disease progresses, MS increase the risk of falls and recurring falls [12]. Moreover, NMS progress slowly and results in disability and greater dependency [13].

The trunk plays an important role in combatting the threats of PD to postural control. Static balance performance and poor gait can be related with trunk muscles in these patients [14]. According to studies, postural instability and falls are more frequent among individuals with worse trunk mobility and axial rigidity [15]. Almost 75% of falls in Parkinson’s Disease occur due to the inability to control the mass of the body during the performance of activities, such as turning around, standing up and bending forward [16].

The beginning of postural instability in PD affects postural control and balance, as it retards the automatic postural responses, primarily due to body rigidity (i.e., poor arm swing during gait, and the tendency of the head to remain aligned with the body during turns) [17].

Managing PD is complex, depending on the stage of the disease and its diverse symptomology. For this reason, therapy must be individualized and adapted to each patient [7]. The physical and therapeutic exercise involved in physical therapy constitutes a key element, along with current medical and pharmacological treatments (e.g., dopamine agonists, levodopa therapy and/or anticholinergic drugs), to improve postural stability, balance and mobility, thereby reducing falls. Levodopa and deep brain stimulation are known to be relatively ineffective at managing the symptoms that affect balance [18]. Therefore, physical therapy is essential and must be undertaken as early as possible. For example, both the improvement of maximum excursion (i.e., forward, right and left directions) and decreased axial rigidity may increase the ability of participants to adopt effective anticipatory strategies to avoid falls and have a positive impact on balance performance [7,16,19,20,21].

The upper part of the body is responsible for two thirds of body weight and its centre of gravity [22]. Other authors mention that, mechanically, the trunk represents almost 50% of body mass, and that head and trunk control is essential to maintain balance during dynamic tasks [23]. Notwithstanding, most studies on physical therapy treatments for PD traditionally focus on the muscles of the lower limb, whereas those addressing the neuromuscular control of central segments are scarce [24,25,26,27]. It has been reported that the strength of lower limb muscles is predictive and related to independence in the activities of daily living, and the preservation of lumbar lordosis may be one of the factors necessary to maintain the efficient biomechanics of these muscles [28]. In a recent study [29], patients with early-stage PD had a deficit of about 20% in force production in lower limb muscle groups compared with age-matched neurologically normal controls. In addition, there is a relative lack of research that aims to understand the deficits related to trunk musculature and its influence on the risk of patients suffering falls. There is an urgent need to evaluate the effectiveness of therapeutic interventions focused on this area and the impact of these treatment techniques on postural stability, looking for conclusive answers across the disease spectrum [21]. Although several authors have carried out treatments aimed at the trunk, whether the focus was on mobility, stretching, endurance or strengthening of the trunk (e.g., Global Postural Re-education; Mezieres physiotherapy; proprioceptive training), no studies have delved into the efficacy of these interventions and their benefits related to balance among PD patients [21,22,30].

Thus, the objective of this review is to determine the effectiveness of specific trunk exercises, as well as their benefits and impacts on static and dynamic balance among idiopathic PD patients.

## 2. Materials and Methods

A systematic review was carried out in February 2022, following Preferred Reporting Items for Systematic Reviews and Meta-analyses (PRISMA) standards [31]. It was registered in the International prospective register of systematic reviews PROSPERO database (CRD42022322063).

An exhaustive search was conducted to find articles published in the last 10 years in five databases (Cochrane Library, SciELO, PEDro, Scopus and PubMed), which were written either in English or Spanish, using the following descriptors: Parkinson’s disease*, “trunk”, “exercise”, “therapy” and “physical therapy”, combined with the boolean operator “AND”.

The following PICOS eligibility criteria were used for the selection of the articles (participants, intervention, comparator, outcomes, study design):

The participants were individuals clinically diagnosed with idiopathic Parkinson’s disease, at stage ≤4 on the Hoehn and Yahr scale, who were also over 18 years of age.

The main intervention consisted of trunk-specific exercises (e.g., strength, elongations, mobility, postural, etc.) carried out alone or integrated with other interventions in the experimental group (EG). The dose, frequency and type of the exercises were not limited.

The selected studies compared the specific trunk exercises (EG) with the control group (CG), which took part in any type of intervention based on other parts of the body but the trunk, or no therapy. The primary outcomes were static and dynamic balance; secondary outcomes were motor status, gait and falls.

Regarding the study type, all were randomized controlled trials (RCTs).

Inclusion criteria: RCTs between 2012 and 2022. It is important to search for the most recent evidence for specific trunk exercises in PD, particularly those studies published during the 10 years prior to the protocol for this systematic review, as well as including important outcomes in relation to balance dysfunction. Those articles were accepted in which the EG conducted specific trunk exercises with or without complementary therapy in the cited body segment.

Exclusion criteria: Observational or descriptive studies. Not written in English or Spanish.

Table 1 displays the results of the searches conducted and the selected articles.

Two independent authors (RLL and SVT) selected the titles and abstracts of the articles that fulfilled the inclusion and exclusion criteria. When discrepancies arose between the researchers, a third party was consulted (MVM). Finally, the characteristics from each study were extracted independently.

The assessment of the methodological quality of the articles included was carried out using the PEDro scale [39].

The meta-analysis was performed using Review Manager (RevMan v.5.3; Cochrane Collaboration, Oxford, UK). Changes from the pre-to post-intervention assessment were obtained directly from the study results. When the data presented in the studies were insufficient for meta-analysis purposes (e.g., means and standard deviation were not provided), the trial authors were contacted for additional data. When authors did not provide standard deviations but did provide *p*-values or 95% confidence intervals, the Review Manager calculator was used to calculate them [40].

Continuous outcomes were analysed using standardized mean differences because all scales were assumed to measure the same underlying symptom or condition, but some studies measured outcomes on different scales [41,42]. For all outcomes, 95% confidence intervals were calculated. The scoring of the different scales was converted so that all scores were in the same direction.

Statistical heterogeneity was examined using I^2^. In addition, we searched for outlier studies using a visual inspection of forest plots. The I^2^ value can be interpreted as the proportion of the total observed variation between studies that can be explained by differences between studies and not by sampling error [41,43]. When the meta-analysis was considered homogeneous, i.e., with an I^2^ value of less than 75%, a fixed-effect model was used. Otherwise, it was considered a heterogeneous meta-analysis and a random-effects model was used. Statistical significance was set as *p* < 0.05, indicating that the effects differed significantly between the intervention and control groups [44]. Furthermore, sensitivity analyses were performed, and sources of heterogeneity were explored by excluding trials with a high risk of detection or attrition bias [40].

The meta-analysis used the following centre of pressure (COP) variables in two different postural conditions (double-leg stance with eyes open and eyes closed): sway area, and total, medio-lateral (ML) and anterior-posterior (AP) path lengths and velocities. To improve the statistical power within the meta-analysis, the length and velocity variables were combined, as these are two mathematically analogous variables due to constant trial times within the studies [45].

## 3. Results

**Figure 1:**
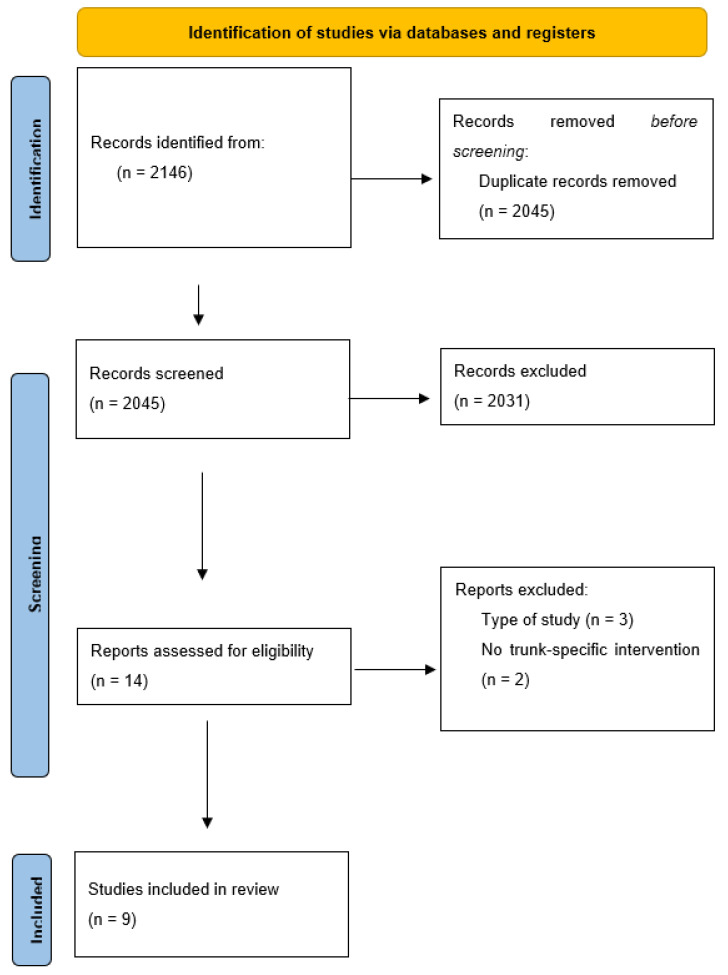
PRISMA flowchart of article selection process.

Having conducted the search, 2146 items were initially obtained. After eliminating any duplicate articles, 2045 were analysed for their title and abstract. After a first screening of those studies that were considered potentially relevant, a full-text critical reading of 14 articles was performed, paying special attention to the study and treatment type. Finally, a total of nine articles were determined when meeting the objective and criteria proposed for this review (Figure 1). Eight of these were included in the meta-analysis.

This section presents the most relevant information for each RCT in relation to the characteristics of the participants, intervention, variables and results (Table 2).

### 3.1. Participant Characteristics

The study sample ranged from 23 to 44 participants, with a total of 240 analysed (EG: 123 and CG: 117). As for gender, 163 were men and 91 were women, with an age range between 65 and 77.2.

The individuals were diagnosed according to the UK Brain Bank criteria for idiopathic Parkinson’s Disease [16,32,33,34] and/or by clinical diagnosis confirmed by a neurologist using the Hoehn & Yahr scale [14,33,37,38].

### 3.2. Variables (Table 3)

#### 3.2.1. Balance

Both static and dynamic balance were evaluated in eight of the nine articles included using posturography with strength platforms [14,16,32,33,34,37,38]. Significant changes were found for balance in three of the studies [16,32,34].

As for the measurement using the Mini BesTest (MBT) for dynamic balance, the studies by Cabrera [16], Terrens [37] and Gandolfi [33] obtained significant changes in the EG *p* < 0.02, *p* = 0.011 and *p* = 0.017, respectively. In the case of the protocol in Cabrera [16], the participants took part in interventions focused specifically on central stabilization, finding significant improvements in anticipatory, reactive postural control and dynamic gait subscales. The Halliwick aquatic group improved significantly post-intervention [37] and the EG with active self-correction exercises, proprioceptive feedback and trunk stabilization exercises plus functional tasks [34].

#### 3.2.2. Mobility

Mobility was generally measured using Timed Up and Go (TUG), obtaining conflicting results [32,34,35,36]. The cohort assessed in one study [34] generally comprised early-stage PD patients who presented with mild to moderate motor symptoms.

#### 3.2.3. Gait

Gait was evaluated by different analysis systems, including movement analysis, cameras, platforms and markers attached primarily to the trunk and lower limbs [14,32,34].

#### 3.2.4. Falls

Falls, balance confidence and self-perception upon falling were assessed using two self-efficacy instruments: the Activity-Specific Balance Confidence Scale (ABC Scale) and the modified Falls Efficacy Scale (mFest) [16,34,37].

#### 3.2.5. Motor Status

Motor status was evaluated in all the studies using the Unified Parkinson’s Disease Rating Scale (UPDRS III) [32,33,34,35,36,37,38] or the Movement Disorder Society-UPDRS (MDS-UPDRS III) [14,16]. The MDS-UPDRS is a revision of the UPDRS originally developed in the 1980s. The MDS-UPDRS was developed to evaluate various aspects of Parkinson’s disease including non-motor and motor experiences of daily living and motor complications [46]. Two articles [34,35] showed no changes in motor status.

**Table 3:** Results of the variables: mobility, gait, balance.

Author, Year	Mobility/Motor Symptom	Gait	Static and Dynamic Balance
Vasconcellos [14] 2021	Movement Disorders Society—Unified Parkinson’s Disease Rating Scale III (MDS-UPDRS-III) observed a reduction of trunk flexion after trunk exercises.	No significant time × group interaction was observed: velocity, hip extension, knee and ankle ROM; with no intragroup differences.	This study failed to find any significant changes in the results of the groups that engaged in exercises at home, unsupervised by a physical therapist.
Cabrera [16] 2020	There were no significant differences between groups in MDS-UPDRS-III Scale (*p* = 0.083).	Significant improvements were found in the anticipatory, reactive postural control, and dynamic gait subscales (*p* < 0.05). The number of falls in the previous month for the EG significantly decreased (*p* = 0.047).	The participants in the EG performed significantly better than those in the CG in the dynamic balance assessment (*p* = 0.002). The EG had a significant improvement in maximal excursion of COP in forward (*p* = 0.048), right (*p* = 0.046) and left (*p* = 0.010) directions of limits of stability.
Terrens [37] 2020	They improved the results in both intervention groups (aquatic and land), without being statistically significant, using the UPDRS-III Scale.	They found no significant changes using modified Falls Efficacy Scale.	No significant differences with Berg balance scale among the three groups compared (Halliwick aquatic exercises; traditional aquatic and land physiotherapy).
Youm [32] 2020	With Timed Up and Go (TUG) significant results were found (*p* = 0.004) (intergroup). They evaluated the participants with the Functional Fitness Test (FFT), obtaining favorable results in 2-minute step test (*p* = 0.044).	With the sit-to-walk test, they obtained improvements in EG in increased length and speed in the first step phase (*p* = 0.003, *p* = 0.006, respectively) and during the second step phase in comparison to the CG (*p* = 0.020 and *p* = 0.028, respectively).	Significant changes were found for anteroposterior speed (AP) (*p* = 0.030) and middle lateral (ML) (*p* = 0.028) of COP trajectory compared with CG
Gandolfi [33] 2019	They obtained significant results in both groups before and after the treatment (*p* = 0.01) but did not find differences between the groups, using the Unified Parkinson’s Disease Rating Scale III.	The number of falls in the previous month for the EG significantly decreased (*p* = 0.004).	Mini BesTest for dynamic balance obtained significant changes in the EG (*p* = 0.017).
Hubble [34] 2019	They failed to obtain significant differences with Timed Up and Go (TUG). No intervention led to a significant change in mobility, motor symptom severity, or freezing of gait at the 12- or 24-week time points.	Step-by-step gait asymmetry analysis with accelerometer obtained favorable results in the EG in vertical head movements and AP (*p* = 0.009 and *p* = 0.011), in trunk AP (*p* < 0.001), and 12 days following the intervention compared to the initial assessment.	Significant changes were identified in the COP elliptical swing area after 12 weeks of intervention and following 24 weeks (*p* = 0.029 and *p* = 0.013 respectively), besides in the ML swing patterns at 12 (*p* = 0.042) and 24 weeks (*p* = 0.043).
Hubble [35] 2018	They failed to obtain significant differences between groups (education or exercise) with TUG or UPDRS-III.	Trunk-specific exercises may improve (or maintain) step-to step symmetry of trunk movements and trunk muscle function in this population.	No significant differences with ABC scale among the groups.
Paolucci [38] 2017	They obtained significant results in the EG (*p* = 0.002) and CG (*p* = 0.012) using the 6-minute walk test. They only obtained improvements in the EG using the UPDRS-III (*p* = 0.007) at 𝑇1.	This study used the Functional gait assessment (FGA) to measure balance during gait, obtaining significant results for the EG, which followed the Mezieres Method (*p* < 0.001), and the CG (traditional exercises at home) (*p* = 0.001).	Berg balance scale (BBS), proved to be statistically significant with respect to the CG (without treatment) in favor of the EG (trunk resistance and stretching exercise program), and remaining so following the 12-week evaluation period (*p* < 0.001).
Capecci [36] 2014	All treated patients, independent of treatment group, showed a significant improvement in the trunk posture in both the sagittal and coronal planes, with respect to baseline.	TUG improvements also seem to positively affect functional gait speed (*p* = 0.028). This benefit is significant when compared with untreated patients.	BBS proved to be statistically significant (*p* < 0.0001) with respect to the CG (without treatment) in favor of the EG (proprioceptive and tactile stimulation, combined with stretching and postural reeducation).

EG: Experimental Group; CG: Control Group; COP: center of pressure; ROM: Range of movement; AP: anteroposterior; ML: middle lateral; T1= First assessment; ABC scale: Activity-Specific Balance Confidence Scale; BBS: Berg Balance Scale; FEST: Falls Efficacy Scale; UPDRS-III: Unified Parkinson’s Disease Rating Scale; MDS-UPDRS-III: Movement Disorders Society—Unified Parkinson’s Disease Rating Scale III; TUG: Timed Up and Go; FFT: Functional Fitness test; FGA: Functional Gait Assessment.

### 3.3. Intervention Characteristics

The specific trunk exercises were based on different methods, such as Halliwick aquatic exercises, the Mezieres method or Global Postural Re-education (Table 4). More specifically, the exercises focused on musculature strengthening [14,32,35], central stabilization training [14,35,36,37], trunk mobility [33,34,35,37], stretching of trunk musculature [32,33,35,36], and postural exercises [33,36,38]. The duration of the sessions varied between four weeks [14,33,36], five to eight weeks [16,38] and 12 weeks [32,33,35,38]. All the interventions were conducted on the ground, except for one carried out in water [37]. The Vasconcellos intervention [14] utilized a remote format supervised by relatives. All participants took part in the interventions in the “ON” status of their medication cycle, and nearly all were guided by a professional physical therapist, except in one case [14].

### 3.4. Methodological Evaluation

Seven of the selected studies [14,16,33,35,36,37,38] featured good methodological quality, with scores between six and eight points on the PEDro scale; two studies [32,34] displayed lower quality (Table 5).

### 3.5. Meta-Analysis

**Figure 2:**
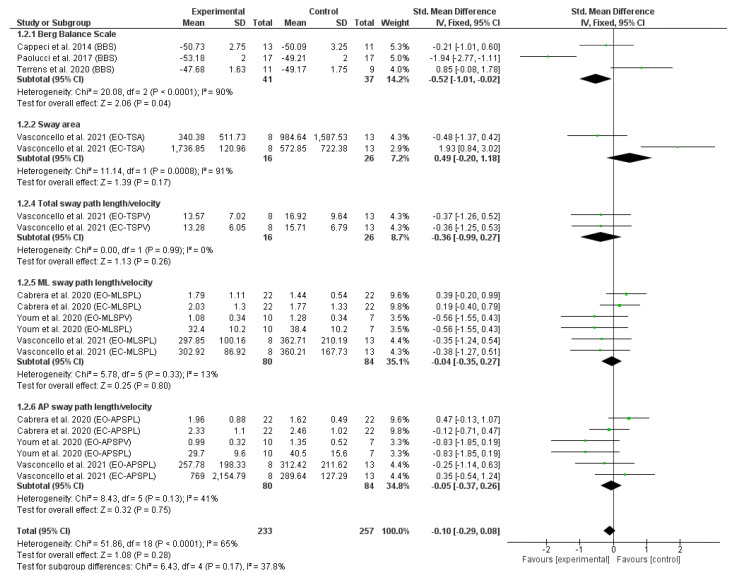
Forest plot of static balance. Displays standardized mean differences (SMD) and confidence intervals (CI) of 95%. References [14,16,32,36,37,38] are cited.

The reporting data from eight RCTs obtained in static and dynamic balance were included in the meta-analysis. The results of the first meta-analysis (Figure 2) revealed the effect of trunk-specific exercises on static balance, measured with posturography [14,16,32] and the BBS [36,37,38]. Previous studies show a moderate relationship between subjective testing and measures of COP displacement, indicating that a combination of subjective and quantitative measures can provide important information that cannot be obtained by separate subjective or quantitative assessment [47].

**Figure 3:**
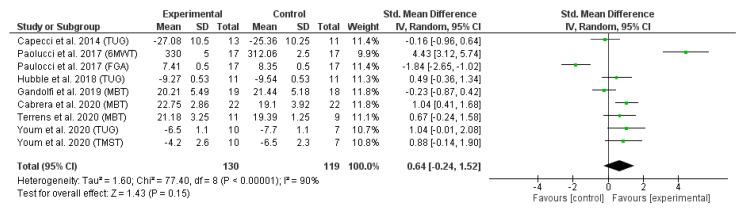
Forest plot of dynamic balance. References [16,32,33,35,36,37,38] are cited.

The results of the second meta-analysis (Figure 3) revealed the effect of trunk-specific exercises on dynamic balance measured using MBT [16,33,37], 6MWT [35], TUG [32,35,36], FGA [38] and TMST [32]. All studies that did not provide the necessary static or dynamic balance data to perform the meta-analysis (means and standard deviations) and for which no response was received from the authors were excluded.

Figure 2 depicts the forest plot of the static balance meta-analysis. Due to the statistical heterogeneity of the results (I^2^ = 65%, *p* < 0.001), a statistical fixed effects model was applied. Patients’ static balance was not significantly improved in the EG in comparison with CG (SMD = −0.10, 95% CI = −0.29, 0.08; *p* = 0.28). However, significant differences were found in favour of EG in static balance measured by a subjective measure using the BBS (SMD = −0.52, 95% CI = −1.01, −0.02; *p* = 0.04).

Figure 3 depicts the forest plot of the dynamic balance meta-analysis. Due to the statistical heterogeneity of the results (I^2^ = 95%, *p* < 0.001), a statistical random effects model was applied. Patients’ dynamic balance was not significantly improved in the EG in comparison with CG (SMD = 0.64 95% CI = −0.24, 1.52; *p* = 0.15).

## 4. Discussion

This systematic review evaluates the efficacy of trunk-targeted exercise and improvements in balance and mobility in PD persons, motivated in part by the current scarcity of research directly assessing this potential link. The results of the meta-analysis suggest that this intervention can generate changes in static balance measured by the BBS with respect to the CG (SMD = −0.52, 95% CI = −1.01, −0.02; *p* = 0.04); however, these changes are not significant nor greater than other interventions if measured with other scales (SMD = −0.10, 95% CI = −0.29, 0.08; *p* = 0.28). As for other aspects, upon comparing the dynamic balance parameters on the pooled effect, no significant differences are found among the groups, albeit the results clearly show that trunk-targeted exercises in the EG are superior to the conventional therapy in the CG in most studies (SMD = 0.64 95%; CI = −0.24, 1.52; *p* = 0.15).

It has, thus, been explained that balance measurements play a key role among this population, since patients with PD tend to have worse balance and mobility due to flexed truncal posture [48], with greater AP and ML displacement of COP than the unaffected population [49,50]. Moreover, it has been highlighted that there are several factors that predispose these patients to falls, such as axial rigidity and postural instability, among others [51]. Certain studies suggest that the static posturography used as an assessment tool proves to be less sensitive than dynamic techniques in distinguishing between patients who suffer falls and those that do not, as these individuals with PD tend to reduce the movement and speed of the trunk when walking to increase their postural control [52]. It is worth highlighting that concomitant deficits in muscle strength means that people with PD would be more likely to experience premature muscle fatigue, which has been shown to significantly impair balance control during standing balance assessments [53]. Furthermore, these deficits in trunk mobility and strength affect much more than balance control in people with PD, as these individuals are also less stable during gait and less capable of performing common activities in daily life (ADL) (e.g., rising from a chair, negotiating stairs) [23].

The clinical impact of falls is considerable, which often leads to an incapacitating fear of suffering new falls that causes patients to consciously limit their own mobility [51,54]. Therefore, the objectives of this review proposed that if therapy that targets trunk segments could increase the functional capacity and both the static and dynamic balance of patients, it will increase their confidence [16,35] and decrease their fear of falling [37]. Indeed, as the scientific literature has stated, by providing these individuals with effective anticipatory strategies, falls are avoided [16,37].

Vasconcellos’ study [14] indicates that trunk-targeted exercises conducted remotely and without the supervision of a therapist fail to provide benefits that improve gait or balance, and there is a reduced adherence to complying with the treatment. Contrary to Vasconcellos’ findings [14], Lee [55] and Gandolfi [33] observed a reduction in trunk flexion after these exercises. In addition, Hubble [34] demonstrated that trunk-specific exercises are associated with improvements in select measures of quiet-standing balance under challenging sensory conditions in individuals with mild to moderate PD severity after 12 weeks. However, there were no improvements in measures of mobility, balance confidence, symptom severity, disability, or quality of life. Previously, in 2018, this same author supported the hypothesis that trunk-specific exercises may improve or, at the very least, maintain the step-to-step symmetry of trunk movements and trunk muscle function in PD [35].

In general, these studies suggest that endurance, mobility and trunk-stretching exercises may be an alternative that favours not only gait symmetry, as they influence trunk movements [35] with statistically significant differences, but also step length and speed [32]. Bestaven [56] applied trunk-targeted exercises over 4 months to hospitalised patients, along with deep brain stimulation treatment, obtaining similar, very positive results in cinematic gait measures, in cadence, step length and speed. Some articles suggest a greater potential to effectively carry out ADLs, such as walking, reaching or moving objects, thereby decreasing the probability of falls [16,32].

Moreover, the study by Rafferty [57] applied a long-term (24 months) treatment protocol with progressive strengthening exercises for the trunk and limbs. It obtained beneficial results in both fast gait speed (without medication) and cadence (with and without medication). The Terrens protocol [37] utilized the Halliwick method as an aquatic therapy focused on central stabilization, as did the study by Volpe [58]. Both obtained significant results for the assessments with UPDRS-III, BBS, the ABC scale, TUG and FES, and postural alterations in the sagital and coronal planes. Other authors have indicated that hydrotherapy could be a safe and effective alternative for patients with problems in the axial axis and chronic diseases [59].

As for patient motor status, measured with the commonly used UPDRS III subscale, it was evaluated in five of the studies in this review [33,34,35,37,38], four of which found statistically significant differences in favour of the EG. Gandolfi [33] showed a three-point decrease in the UPDRS III after a month of follow-up, as did the studies by Paolucci [38] and de Lena [60], in which exercises were applied targeting posture, strengthening, mobility, trunk stretching and central stabilization, obtaining significant changes in the scores of 90% of participants. Several protocols focused specifically on exercises targeting body and movement awareness, which had already proved to improve postural anomalies [36,38,56,58].

Additionally, it has been demonstrated that progressive exercises targeting improvements in the function of the deeper trunk muscles provide a safe and inexpensive exercise-based therapy option that helps to maintain and/or improve postural stability and, ultimately, contribute to improving quality of life for people with PD [61]. The range of movement for trunk flexion, extension and rotations in mild–moderate PD patients was correlated with health-related quality of life assessed with the Quality of Life in Parkinson’s Disease-39 and EuroQol-5D questionnaires [62]. Finally, physiotherapy centred on rachis mobility improves the quality of gait and reduces the number of falls in patients with PD. Dealing with these axial symptoms is a challenge that not only concerns improving the autonomy and quality of life of these patients, but also reducing the cost of medical and health services [53].

Among the limitations of this review is the small sample size of the articles included, which complicates the extrapolation of the results. The PEDro scale indicates a deficit in the blind process of certain studies, nearly all of which applied a single-blind trial (of the evaluator). Future studies should include larger sample sizes with follow-up studies (short intervention can lead to ineffective outcomes) and explore whether trunk exercise can benefit people with more advanced Parkinson’s disease. Furthermore, the studies were heterogeneous in the intervention protocols and measurements of results. The strength of this review lies in the methodological quality of its RCTs, since most of them (77.78%) obtained a score between 6 and 8 on the PEDro scale.

As for the practical implications, the benefits obtained through trunk stability training were in general higher than those achieved through non-specific exercise, offering clinical benefits that could have an economic impact, by avoiding falls and their costly expenses. Nevertheless, it is necessary to continue work in this line of research focused on exercises related to central stabilization, trunk strengthening and mobility, in order to verify their long-term effects and at more advanced stages of the disease [63].

## 5. Conclusions

Although the specific trunk exercises failed to show significant differences in all the variables that measured static and dynamic balance, it is worth noting that the studies included in this systematic review are committed to the use of these exercises as a complement to conventional treatment, as they have been observed to have a considerable impact on both balance and central mobility in PD patients. However, more studies need to be conducted, ones with detailed treatment protocols and similar results measurements to support the effectiveness of this intervention on balance and motor status in PD in the short and long term.

## Figures and Tables

**Table 1:** Search strategies in the different databases.

Databases and Search Terms	Results	Selected Articles
**Cochrane Library** Parkinson’s Disease* AND “physical therapy” Parkinson’s Disease* AND “trunk” Parkinson’s Disease* AND “physical therapy” AND “trunk” Parkinson’s Disease* AND “therapy” AND “trunk” Parkinson’s Disease* AND “Exercise” AND “trunk”	292 118 22 61 61	Vasconcellos et al. [14] 2021 Youm et al. [32] 2020 Gandolfi et al. [33] 2019 Hubble et al. [34] 2019 Hubble et al. [35] 2018 Capecci et al. [36] 2014
**SciELO** Parkinson’s Disease* AND “physical therapy” Parkinson’s Disease* AND “trunk” Parkinson’s Disease* AND “physical therapy” AND “trunk” Parkinson’s Disease* AND “therapy” AND “trunk” Parkinson’s Disease* AND “Exercise” AND “trunk”	33 5 1 1 0	
**PEDro** Parkinson’s Disease* AND “physical therapy” Parkinson’s Disease* AND “trunk” Parkinson’s Disease* AND “physical therapy” AND “trunk” Parkinson’s Disease* AND “therapy” AND “trunk” Parkinson’s Disease* AND “Exercise” AND “trunk”	56 24 3 5 14	Vasconcellos et al. [14] 2021 Youm et al. [32] 2020 Gandolfi et al. [33] 2019 Hubble et al. [34] 2019 Hubble et al. [35] 2018 Paolucci et al. [36] 2017
**Scopus** Parkinson’s Disease* AND “physical therapy” Parkinson’s Disease* AND “trunk” Parkinson’s Disease* AND “physical therapy” AND “trunk” Parkinson’s Disease* AND “therapy” AND “trunk” Parkinson’s Disease* AND “Exercise” AND “trunk”	948 644 30 135 67	Vasconcellos et al. [14] 2021 Cabrera et al. [16] 2020 Terrens et al. [37] 2020 Youm et al. [32] 2020 Gandolfi et al. [33] 2019 Hubble et al. [34] 2019 Hubble et al. [35] 2018 Paolucci et al. [38] 2017 Capecci et al. [36] 2014
**PubMed** Parkinson’s Disease* AND “physical therapy” Parkinson’s Disease* AND “trunk” Parkinson’s Disease* AND “physical therapy” AND “trunk” Parkinson’s Disease* AND “therapy” AND “trunk” Parkinson’s Disease* AND “Exercise” AND “trunk”	255 411 5 44 20	Vasconcellos et al. [14] 2021 Cabrera et al. [16] 2020 Terrens et al. [37] 2020 Youm et al. [32] 2020 Gandolfi et al. [33] 2019 Paolucci et al. [38] 2017 Capecci et al. [36] 2014

**Table 2:** Brief description of articles selected.

Author, Year	Type of Study and Participants	Characteristics of the Intervention	Measured Variables	Main Results
Vasconcellos [14] 2021	RCT 28 participants EG: 14 CG: 14	EG: Trunk exercise program and pelvic floor muscles. CG: Upper and lower limb exercises. Both groups performed home-based exercises under caregiver supervision. Protocol described by Silva and Motta. Duration: 3 times/daily/3 weeks.	Static balance (stabilometry), using a force platform connected to an external amplifier and a motion analysis system. Gait evaluated using the Qualisys^®^ movement analysis system.	No intragroup differences were observed for the COP range (*p* = 0.353), COP velocity (*p* = 0.318) or gait measurements (*p* = 0.778). Trunk-strengthening exercises failed to improve gait and balance compared to limb exercises. A total of 33% failure to complete treatment (9 individuals). The absence of face-to-face therapist supervision may have affected patients’ performance during interventions.
Cabrera [16] 2020	RCT 44 participants EG: 22 CG: 22	EG: 24 sessions core stabilization training program. CG: active joint mobilization, muscle stretching and motor coordination exercises. Duration: 45 min./day, three times/week/8 weeks.	Dynamic balance with Mini-BesTest and standing balance with posturography using the Nintendo Wii (maximal excursion of COP during the Modified Clinical Test of Sensory Interaction on Balance and the Limits of Stability test); Balance confidence (ABC Scale).	A significant improvement in dynamic balance was observed in the EG compared to the CG (*p* = 0.002); in self-perceived confidence related to balance (*p* = 0.047); and maximal excursion of COP in forward (*p* = 0.048), left (*p* = 0.010), right (*p* = 0.046) between-group differences. A core stability program may influence anticipatory postural adjustments.
Terrens [37] 2020	Pilot Trial 30 participants (G1) 11 (G2) 10 (G3) 9	(G1) Halliwick aquatic exercises (trunk mobility, core stabilization and rotational exercises); (G2) traditional aquatic and (G3) land-based physiotherapy. Duration: 60 min./week/ 12 weeks.	Balance (BBS and Mini BesTest), Falls (mFES), Motor status (UPDRS-III).	No significant differences within groups were found in UPDRS-III, BBS or mFES scores post-intervention for any groups. Halliwick aquatic group improved significantly in the Mini BesTest post-intervention, i.e., promising results for balance (*p* = 0.011).
Youm [32] 2020	RCT 23 participants EG: 12 CG: 11	EG: trunk resistance and stretching exercise program. CG: no intervention. Duration: 60–90 min., 3 times/week, 12 weeks.	Trunk mobility scale (TMS test), FFT, TUG, standing balance test with a platform, and sit-to-walk test with Nexus software.	The EG showed improvements in FFT, trunk mobility, standing balance and dynamic stability compared with the CG (all *p* < 0.05). This 12-week exercise program improved fall-related factors in patients with PD.
Gandolfi [33] 2019	RCT 37 participants EG: 19 CG: 18	EG: Active self-correction exercises (with visual feedback (i.e., mirror), with proprioceptive feedback (EMG feed-back), and without any feedback) + trunk stabilization exercises+ functional tasks (i.e., dual-task exercises). CG: joint mobilization, muscle strengthening and stretching, overground gait-training and balance exercises. Duration: 60 min./5 days/week, 4 weeks.	Forward trunk flexion severity (degree). UPDRS III, dynamic and static balance (Mini BesTest and using an electronic monoaxial platform), pain falls, and quality of life assessment.	The EG reported a significantly greater reduction in forward trunk flexion than the CG from T0 to both T1 (*p* = 0.003) and T2 (*p* = 0.004). The improvements in dynamic and static balance were significantly greater for the EG than the CG from T0 to T2 (*p* = 0.017 and *p* = 0.004, respectively). The four-week trunk-specific rehabilitation training decreased forward trunk flexion severity and increased postural control.
Hubble [34] 2019	RCT 24 participants EG: 13 CG: 11	EG: exercise (trunk strength, endurance, and mobility) and falls prevention education. CG: weekly pack of printed multidisciplinary education materials: health tips about lifestyle (e.g., exercise) and/or condition-related issues (e.g., poor sleep quality). Duration: 90 min./week, 12 weeks.	Motor symptom severity (UPDRS-III), balance confidence (portable force plate and ABC Scale), mobility (TUG), quality of life (39-item Parkinson Disease Questionnaire) and quiet-standing balance.	No significant changes in clinical outcomes following the intervention. During quiet standing, sway area on a foam surface without vision was reduced for the EG at 12 (*p* = 0.029) and 24 weeks (*p* = 0.013). The EG demonstrated reduced sway variability at 12 (*p* = 0.042) and 24 weeks in the medial–lateral direction (*p* = 0.043). No changes in quiet standing balance for the CG.
Hubble [35] 2018	RCT 24 participants EG: 13 CG: 11	EG: falls prevention education + exercises: trunk mobility exercises to improve ROM; endurance and stability of the trunk muscles (multifidus, erector spinae, obliques, transverse abdominus, rectus abdominus); and stretching and walking in a real-world environment. CG: multidisciplinary falls prevention education. Duration: 90 min./week, 12 weeks.	Mobility (TUG), walk (gait analysis, accelerometer), falls (ABC scale), motor symptom severity (UPDRS-III).	Statistically significant and clinically relevant improvements in anterior–posterior step-to-step trunk symmetry (*p* < 0.001) in the EG. CG recorded statistically significant and clinically meaningful reductions in medial–lateral and vertical step-to-step trunk symmetry at 12 weeks (*p* < 0.001).
Paolucci [38] 2017	RCT 36 participants EG: 17 CG: 19	EG: Mezieres method, 3 postures to correct variations in the dorsal curve, perceive the alignment of trunk and promote diaphragmatic breathing. CG: simple home exercise Duration: 1 h, 2 times/week, 5 weeks, 10 sessions.	Balance (BBS) gait balance (FGA), mobility (SMWT) and disease-related disability (UPDRS-III).	In the Mezieres group, the BBS (*p* < 0.001) and trunk flexion test (*p* < 0.001) improved significantly at 𝑇1 and remained the same at 𝑇2. Between groups, significant changes were reported in FGA (*p* = 0.027) and UPDRS Total (*p* = 0.007) at 𝑇1 and in FGA (*p* = 0.03) at 𝑇2. The Mezieres approach is effective in improving the flexibility of the trunk and balance.
Capecci [36] 2014	RCT 24 participants EG: 13 CG: 11	EG: 7 participants, proprioceptive and tactile stimulation, combined with stretching and PR. Six participants had PR as well as Kinesio taping strips applied to their trunk muscles, according to the features of their postural abnormalities CG: No intervention. Duration: 40 min./3 times, 4 weeks, 12 sessions.	Balance (BBS), mobility (TUG) and degrees of trunk bending in the sagittal and coronal planes.	At T1, all treated patients showed a significant improvement in trunk posture in both the sagittal (*p* = 0.002) and coronal planes (*p* = 0.01), compared with baseline. Moreover, they showed an improvement in measures of gait and balance (*p* < 0.01). Benefits persisted at T2 for all measures, except lateral trunk bend. No differences were found when comparing the PR and KT groups.

RCT: randomized clinical trial; EG: experimental group; CG: control group; G: group; min.: minutes; COP: centre of pressure; ABC scale: Activity-Specific Balance Confidence Scale; BBS: Berg Balance Scale; Mini Best-Test: Mini-Balance Evaluation Systems Test; mFEST: modified Falls Efficacy Scale; UPDRS: Unified Parkinson’s Disease Rating Scale (UPDRS-III); TUG: Timed Up and Go; FFT: functional fitness test; TMS: trunk mobility scale; ROM: range of movement; FGA: functional gait assessment; PR: postural re-education; KT: Kinesio taping; T0 = baseline assessment; T1 = first assessment; T2 = Second assessment.

**Table 4:** Protocols for trunk-targeted intervention in the studies selected.

Types of Treatments	Other Complementary Exercises	Stretching	Postural Rehabilitation	Central Stabilization	Trunk Strengthening	Trunk Mobility
Vasconcelos [14]				**X**	**X**	
Cabrera [16]				**X**		
Terrens [37]	Gait and balance			**X**		**X**
Youm [32]		**X**		**X**	**X**	
Gandolfi [33]		**X**	**X**	**X**	**X**	**X**
Hubble [34]	Education and self-care preventing falls			**X**	**X**	**X**
Hubble [35]	Education and self-care preventing falls			**X**	**X**	**X**
Paolucci [38]	Respiration	**X**	**X**			
Capecci [36]	Kinesio taping	**X**	**X**			**X**

**Table 5:** Evaluation of methodological quality according to the PEDro scale [39].

ITEM (PEDro Scale)	1	2	3	4	5	6	7	8	9	10	11	POINTS
Vasconcellos [14] 2021	X	X	X	X	N	N	X	N	X	X	X	7/10
Cabrera [16] 2020	X	X	X	X	N	N	X	X	X	X	X	8/10
Terrens [37] 2020	X	X	X	X	N	N	X	N	X	X	X	7/10
Youm [32] 2020	N	X	N	X	N	N	X	N	N	X	X	5/10
Gandolfi [33] 2019	N	X	N	X	N	N	X	X	X	X	X	7/10
Hubble [34] 2019	X	X	N	X	N	N	X	N	N	N	X	4/10
Hubble [35] 2018	X	X	N	X	N	N	X	X	X	X	X	7/10
Paolucci [38] 2017	X	X	X	X	N	N	X	X	X	X	X	8/10
Capecci [36] 2014	N	X	X	X	N	N	X	X	X	X	X	7/10

X: the article fulfils the criteria. N: the article fails to fulfil the criteria.

## Data Availability

Not applicable.
